# On the spot: utilization of directional cues in vibrational communication of a stink bug

**DOI:** 10.1038/s41598-018-23710-x

**Published:** 2018-04-03

**Authors:** Janez Prešern, Jernej Polajnar, Maarten de Groot, Maja Zorović, Meta Virant-Doberlet

**Affiliations:** 1 National Institute of Biology, Dept. of Organisms and Ecosystems Research, Večna pot 111, SI-1000 Ljubljana, Slovenia; 2 Present Address: Agricultural Institute of Slovenia, Dept. of Animal Production, Hacquetova ulica 17, SI – 1000 Ljubljana, Slovenia; 3 Present Address: Slovenian Forestry Institute, Dept. of Forest Protection, Večna pot 2, SI – 1000 Ljubljana, Slovenia; 4 Present Address: University of Ljubljana, Medical Faculty, Institute of Pathophysiology, Brain Research Laboratory, Vrazov trg 2, SI – 1000 Ljubljana, Slovenia

## Abstract

Although vibrational signalling is among the most ancient and common forms of communication, many fundamental aspects of this communication channel are still poorly understood. Here, we studied mechanisms underlying orientation towards the source of vibrational signals in the stink bug *Nezara viridula* (Hemiptera, Pentatomidae), where female vibrational song enables male to locate her on the bean plant. At the junction between the main stem and the leaf stalks, male placed his legs on different sides of the branching and orientation at the branching point was not random. Analyses of signal transmission revealed that only a time delay between the arrival of vibrational wave to receptors located in the legs stretched across the branching was a reliable directional cue underlying orientation, since, unexpectedly, the signal amplitude at the branching point was often higher on the stalk away from the female. The plant and the position of the vibrational source on the plant were the most important factors influencing the unpredictability of the amplitude cue. Determined time delays as short as 0.5 ms resulted in marked changes in interneuron activity and the decision model suggests that the behavioural threshold is in the range between 0.3 and 0.5 ms.

## Introduction

Substrate-borne vibrations are among the most ancient and widespread modalities used to gather information present in the environment^1–4^, yet many fundamental aspects underlying behaviour guided by vibrational signals/cues are still virtually unexplored. Since vibrational signalling has been traditionally perceived as a highly specialized and rare form of animal communication, vibrational communication in vertebrates like elephants inevitably captures our attention^5^; however, it is small animals like insects that may provide a crucial insight into life in the vibratory world.

In insects, substrate vibrations play an important role in various behavioural contexts, like finding partners, preys or hosts^6,7^, and for most of these behaviours the successful location of a relevant vibrational source is essential. Accurate location of the source of vibrations has been documented in several insect taxa^8^; however, the underlying processes are poorly understood. The small body size of insects and rapid propagation of vibrations through the substrate impose complex constraints on this communication modality, where localization of a vibration source may be regarded as an interaction between the transmission properties of the substrate and insect’s physiology and behaviour^8–11^. Consequently, to get a proper insight into mechanisms underlying orientation towards the source of vibrational signals, one should analyse the behaviour, identify the potential directional cues and link them to the physical properties of the natural environment, as well as to receptor mechanisms and processing in the central nervous system. While such integrative approach has been applied to studies of insect audition^12^, comprehensive studies on model species relying on vibration communication are still lacking.

Here, we investigated the mechanisms underlying location of a vibration source in the southern green stink bug *Nezara viridula*, which is an ideal model species for integrative studies of mechanisms guiding orientation towards the source of vibrational signals. Previous behavioural studies revealed that males of this species use vibrational song emitted by females to find them on a host plant^13^. A stationary female continuously emits a female calling song (FCS), which triggers male searching behaviour and also provides directional cues. FCS is characterized by narrow-band vibrations with dominant frequencies between 80–120 Hz^14,15^ and the signal production mechanism in this species has been studied^16,17^. Furthermore, transmission of vibrational signals with such frequency characteristics appears to be less complex than transmission of broad-band vibrational signals^18–20^. Physiology of individual sense organs involved in vibratory communication of this species has also been investigated^21^. However, although its body size of 1 cm also allows the neurophysiological studies at the level of the central nervous system^22^, the information on signal integration in higher-order neurons is lacking.

More generally, the problem of orientation on a plant with complex geometry can be broken into a series of one-dimensional rods interspersed with crossings, where the most direct route to the source is in the direction where signals are coming from. The differences in arrival times and in amplitudes of vibrational wave reaching receptors located in the legs positioned on the substrate are the most obvious directional cues^8,23–25^. We therefore combined the analysis of orientation behaviour at branching points on a bean plant (*Phaseolus vulgaris*), which is one of the preferred host plants of *N*. *viridula*, with the analysis of transmission of vibrational signals through this plant species in order to obtain the information on availability, reliability and utilization of directional cues. In the next step, we performed neurophysiological investigations on processing the temporal directional cues in the central nervous system. To support our experimental findings, we also modelled the ratio of possible decisions based on the data on propagation of naturally emitted FCS and compared the results with observed behaviour.

## Results

### Male searching behavior

**Figure 1:**
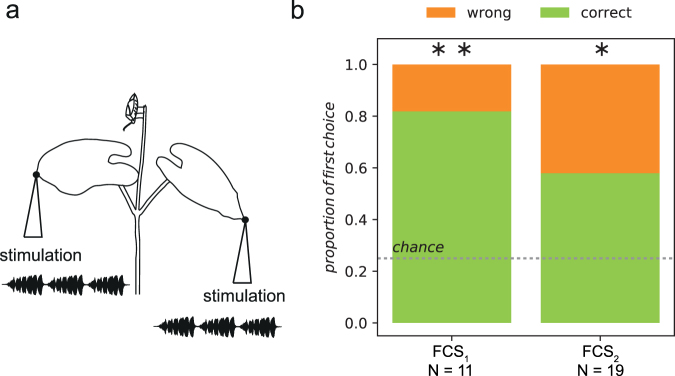
Orientation behaviour of *N*. *viridula* males. (**a**) Schematic presentation of the experimental set-up showing the positions of female calling song (FCS) delivery to the bean plant. The male was placed on the top stem to avoid bias and the side of stimulation was randomly selected. (**b**) Proportions of males making correct or wrong first directional choice at the 4-way crossing. “Chance” indicates the probability of choosing the same branch first at random, i.e. in absence of detectable direction cues (0.25). FCS_1_ and FCS_2_ indicate the first choice with the respective FCS treatments (for details see Materials and Methods). N = number of males tested. Asterisks indicate a significant difference between correct and random choice (Chi-square test, *P < 0.05; **P < 0.01).

The body size of *N*. *viridula* males is around 1 cm and when males are searching for a calling stationary female, they stop at the junction between the main stem and leaf stalks and stretch across the possible paths by placing their legs on different sides of the branching^13^ (Supplementary Video S1). When reaching a junction, a searching male has to choose one of the four available directions and in a random situation (i.e. absence of directional cues) the theoretical probability of choosing each branch is 0.25. Regardless of the FCS used, in playback experiments most males walked first to the stalk of the vibrated leaf (Fig. 1b) and this choice was significantly overrepresented than expected by random orientation (FCS_1_, number of tested males (N) = 11: 81%, *χ*^2^ = 72.02, df = 1, *P* < 0.01; FCS_2_, N = 19: 58%, *χ*^2^ = 42.91, df = 1, *P* < 0.05).

### Propagation of the female calling song

#### Propagation of the naturally emitted FCS

**Figure 2:**
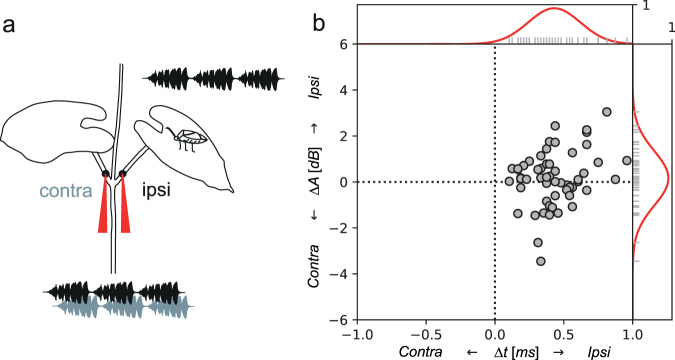
Propagation of naturally emitted female calling song. (**a**) Schematic representation of the setup used to measure signal transmission over the stem – stalk node. *N*. *viridula* female was calling on the leaf and the emitted signals were recorded with two laser vibrometers (red) from the petioles of the two opposite leaves, approximately at the points where a searching male would place legs after reaching the node (see also Supplementary Video S1). (**b**) Time differences (*Δt*) and RMS amplitude difference (*ΔA)* of vibrational signals simultaneously recorded at positions “ipsi” and “contra”. On y axis, values below 0 indicate signals in which RMS amplitude was higher on the contralateral stalk. Number of pulse trains analysed = 55. The bell-shaped curves at the side of the plot represent distributions of the measurements, obtained with convolution of each point by Gaussian unitary curve. These distributions were later used in the behavioural threshold model (Fig. 6).

We recorded the FCSs emitted by 11 females with two laser vibrometers, simultaneously from stalks at the opposite sides of the stem-stalk junction, where the distance between the recording points corresponded to the male leg span (Fig. 2a). The time delay (*Δt*) in arrival of vibrational pulse train to the ipsilateral and contralateral leaf stalk (labelled relative to the position of the calling female) was determined by cross-correlation function (see Materials and Methods) and, predictably, pulse trains were registered earlier on the ipsilateral stalk (*Δt*, mean ± SEM = 0.41 ± 0.16 ms) (Fig. 2b).

The measurements revealed the average RMS amplitude difference (*ΔA*) of 0.16 ± 0.91 dB (mean ± SEM) between two stalks; however, in 38% of recordings the calculated RMS amplitude was higher on the contralateral side (Fig. 2b). Moreover, there was no consistency among measurements and, even when a female was not moving, the side of higher amplitude often changed from one pulse train in a calling bout to another.

#### Two − dimensional characterization of FCS propagation

**Figure 3:**
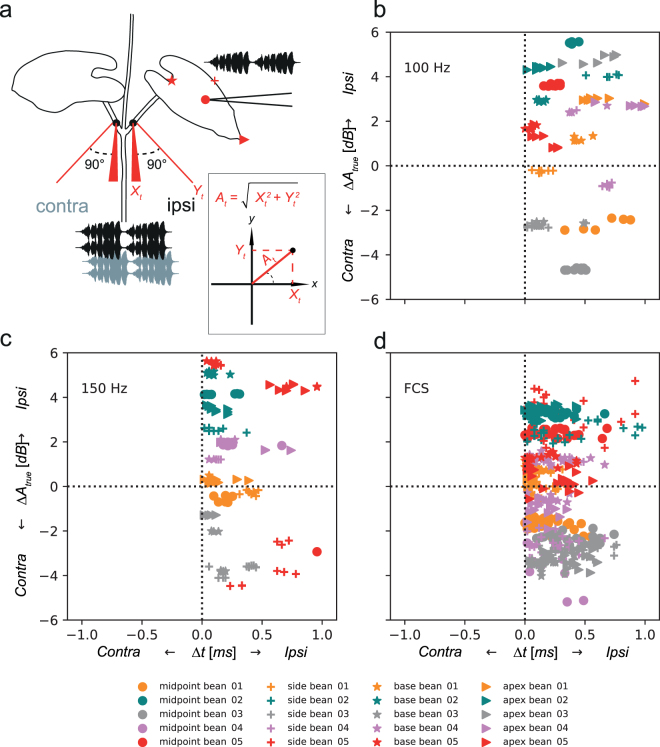
Two−dimensional characterization of female calling song propagation. (**a**) Schematic representation of a recording set-up with two laser vibrometers per stalk positioned perpendicularly to each other (red). Inset illustrates how true amplitude (*A*_*true*_) at each position on the stalks was calculated from amplitude information (*Y*_*t*_) and (*X*_*t*_) from two perpendicularly aligned laser vibrometers. Stimuli 100 Hz, 150 Hz or pre-recorded FCS were applied to the leaf at different positions (midpoint, side, base and apex) and symbols (**a**) match the symbols in (**b**,**c**,**d**). (**b**,**c**,**d**) Time differences (*Δt*) and calculated true RMS amplitude difference (*ΔA*_*true*_*)* of vibrational signals simultaneously recorded at positions “ipsi” and “contra” when different stimuli were used. Values below 0 indicate signals in which true amplitude was higher on the contralateral stalk. For more detailed information, see text.

The amplitude measurements along single axis, as applied above, may not accurately describe the motion of the stem in two-dimensional plane^11,26^ and the inconsistency of *ΔA* may result from underestimation of true amplitude of vibration. In order to calculate the true amplitude of vibration on the stalks, we registered vibrational signals from the ipsilateral and contralateral stalk simultaneously with four laser vibrometers, at each position by two laser vibrometers positioned perpendicularly to each other (Fig. 3a) (see Materials and Methods). Vibrational signals (100 Hz, 150 Hz and FCS) were delivered to the plant at four different locations on the leaf (Fig. 3a). Regardless of where on the leaf we applied the stimuli or which stimulus was used, *Δt* again provided reliable directional information, while *ΔA*_*true*_ was revealed as an unreliable cue (Fig. 3b–d). Vibrations were always registered earlier on the ipsilateral stalk (*Δt* mean ± SEM = 0.25 ± 0.09 ms). The true RMS amplitude difference (*ΔA*_*true*_) between the left and right stalk was 1.58 ± 1.46 dB (mean ± SEM); however, overall in 40% of recorded signals (317/790) the calculated real amplitude was higher on the contralateral stalk.

Machine-learning procedures (RandomForestClassifier with bootstrap sampling, see Materials and Methods for details) were used to estimate the relative importance of different parameters (plant, stimulation point, stimuli) on reliability of *ΔA*_*true*_ as a directional cue. The plant was revealed as the most important factor (relative score, mean ± SD = 0.74 ± 0.06), followed by the position of the stimulus delivery (0.21 ± 0.06), while the stimulus itself received the lowest score (0.05 ± 0.04).

### Interneurons responding to the time delay in arrival of vibrational signal to different legs

**Figure 4:**
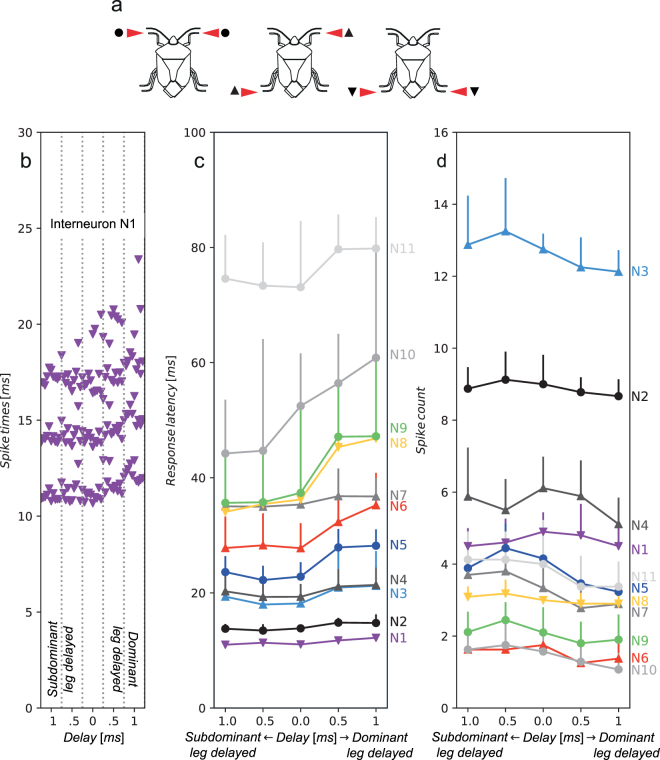
Response of eleven interneurons to time delay in onset of leg stimulation. (**a**) Schematic presentation of leg stimulation configurations (left prothoracic/right prothoracic; left metathoracic/right prothoracic; left metathoracic/right metathoracic). Symbols in (**a**) indicating leg stimulation configurations match those in (b–d). (**b**) Responses of interneuron N1 to applied time delay between leg stimulation. N1 responded with three spikes per spike train, visible as three distinct clusters. The spike pattern changes when stimulation of dominant leg was delayed. (**c**) Changes in the response latency of 11 interneurons. (**d**) Effect of the applied time delay on the spike count. In (b–d) same colours and same symbols indicate the same neuron. Neurons, represented by black and grey colours were not successfully stained.

Since propagation measurements revealed time difference as an available and reliable directional cue, we investigated whether biologically relevant time difference (0.5–1 ms) is detected and processed in the central nervous system of *N*. *viridula*. We recorded single neurons in the prothoracic and central ganglion while stimulating two legs, one on each side of the body, in different combinations (Fig. 4a). Among 51 neurons recorded in 42 individuals, in 11 interneurons neuronal activity was influenced by the time delay in the onset of the stimulation of the two legs (Fig. 4c,d).

These neurons were labelled N1 to N11, according to the increasing response latency when both legs were stimulated simultaneously. The response latencies 11–73 ms indicate that the recorded nervous cells belonged to interneurons at different levels of processing of vibrational information in the central nervous system. In all 11 interneurons, the impact of the time delay between the onsets of stimulation of individual leg was evident in change of spike count or in the change of the response latency measured from beginning of the stimulation, or both (Fig. 4c,d). In all of them, a delay in stimulation of one of the legs had greater impact on neuronal response compared to the reversed situation. We labelled the leg with greater impact as dominant and accordingly, the other leg as subdominant.

In all 11 interneurons, the stimulation of the dominant leg delayed for 0.5 ms resulted in increased response latency varying between 6% in N7 and 26% in N9; in the latter response latency increased from 37 ms to 47 ms (Fig. 4c). Delayed stimulation of the dominant leg decreased the mean spike count up to 29% (from 1.75 to 1.25 in N6) (Fig. 4d). In contrast, the observed effect of delayed stimulation of the subdominant leg differed and was expressed as increase or decrease in the response latency and as reduced or increased number of spikes. The effect was particularly striking in N10, where the time delay at the dominant and subdominant leg had opposite effects; while the delay at latter resulted in reduced response latency and increased spike count, the delay at former increased response latency and reduced the spike count. Although the mean response latency of neurons N7–N9 was very similar when legs were vibrated simultaneously, the observed effect of the time delay differed among them, potentially due to different configuration of leg stimulation. The applied time delay often resulted also in changes in response latency variability (Fig. 4c).

**Figure 5:**
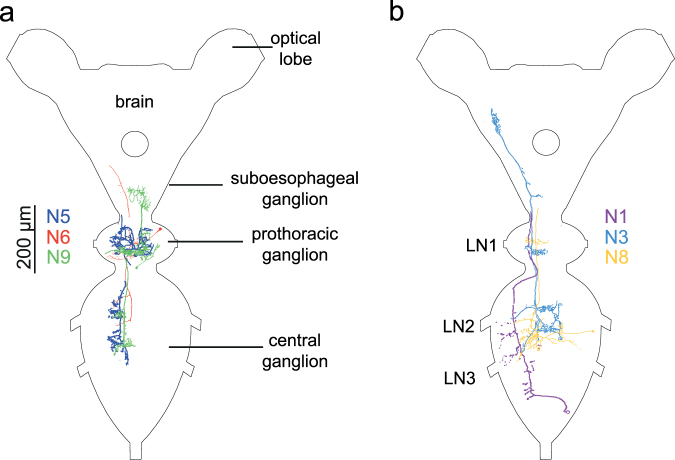
Morphology of interneurons responding to time delay in onset of leg stimulation. (**a**) Interneurons with somata in the prothoracic and (**b**) in the central ganglion. LN1-3, leg nerves 1-3. Note beaded dendrites in some of the stained neurons. The colours match those in Fig. 4. Neurons N2, N4, N7, N10 and N11 shown in Fig. 4 were not successfully stained.

Most of the interneurons were recorded from the prothoracic ganglion, with the exception of N1 and N8, which were recorded from the central ganglion. We successfully stained six of 11 interneurons mentioned above (Fig. 5). All six stained neurons were following the same basic principle. In the ganglion, which contained neurons’ soma, local connection was made over the midline with the contralateral side. Posterior connections followed the contralateral side in all six interneurons; similarly, anterior connections followed the contralateral side with the exception of N9, which continued upwards on the ipsilateral side.

### Behaviour threshold – model predictions

We used the model to evaluate the range of behavioural (sensory) thresholds for both time delay and RMS amplitude difference. The model’s inputs were proportions of time delays and amplitude differences from the complete pool of measured values obtained in propagation measurements of the naturally emitted FCS (Fig. 2b). For the purpose of interpretation and modelling directional choice, we labelled the signals containing cues, which enable correct orientation decision (i.e. earlier signal onset or higher amplitude on the position ipsilateral to the vibrational source) “correct” and the opposite “misleading”, referring to the perspective of a searching male.

**Figure 6:**
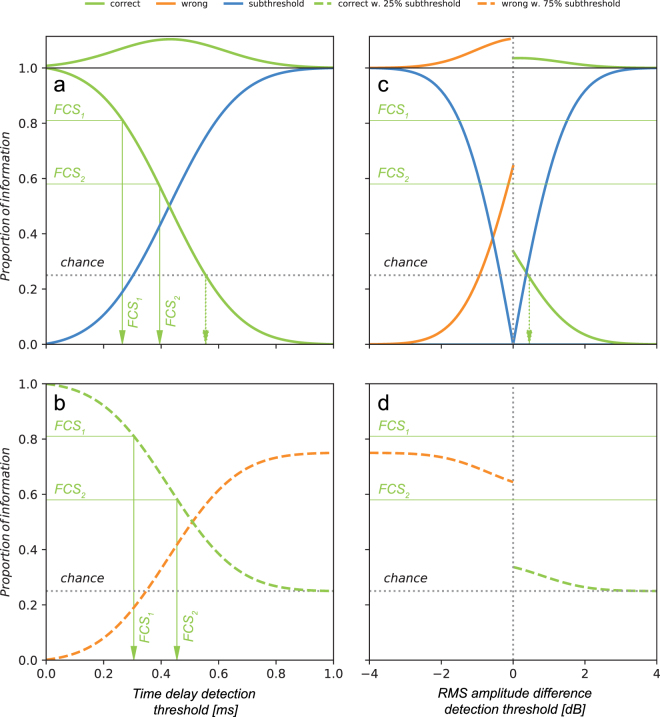
Behavioural threshold model. The Gaussian fit on the distribution of measured parameters shown in Fig. 2 (a,c; top panels) is displayed as a function of the theoretic behaviour threshold and the proportion of measured values above this threshold (a,c; bottom panels). Proportion of ambiguous, sub-threshold values (a,c; solid blue lines) increases away from the threshold 0, inversely to above-threshold values. The latter are distinguished according to sign: those indicating ipsilateral (“correct”) side of the crossing are termed correct (green lines) and those indicating contralateral (“wrong”) side are termed misleading (orange lines). Under the assumption that the male makes a random choice when receiving sub-threshold cues, the ambiguous proportion was added evenly to correct or misleading cues in the second model run: ¼ to correct and ¾ to misleading cues (b,d; dashed lines), reflecting the fact that measurements were only done on two branches of a four-way crossing and assuming that parameters on non-measured contralateral branches were identical to the measured contralateral branch. (**a**,**b**) Time delay (*Δt*). No misleading values were obtained with direct signal measurements. According to the model (**a**), 81% of correct first choices in behavioural experiments with FCS_1_ correspond to 0.27 ms threshold and 58% of correct first choices with FCS_2_ to 0.39 ms (vertical arrows). When the measured correct cues were augmented with 25% of ambiguous values in the second model run (**b**), those choices corresponded with 0.39 ms and 0.46 ms thresholds, respectively. (**c**,**d**) RMS amplitude difference (*ΔA*). 62% measurements contained correct cues and 38% contained misleading cues. However, assuming again that parameters on non-measured branches were identical to those on the measured contralateral branch, the proportion of misleading cues has to be tripled, resulting in 35% of correct and 65% of misleading cues (**c**). Thus, making a directional choice better than chance can only be achieved at threshold below 0.45 dB (vertical arrow), and never better than 35%. The “misleading” proportion was further increased when ambiguous values were added (**d**).

Green line in top panel of Fig. 6a shows the distribution of measured time delay values from signal propagation experiments. The proportion of correct cues (Fig. 6a - solid green line) decreased and the proportion of sub-threshold cues increased inversely (Fig. 6a - solid blue line) with the increase of sensory threshold. Using values from behavioural trials and under the assumption that the male does not orient when directional cues are below the sensory threshold, the first model suggests that behavioural threshold is as low as 0.27 ms for FCS_1_ and 0.39 ms for FCS_2_. On the other hand, under the assumption that searching males randomly choose one of the available directions when they encounter signals containing sub-threshold directional cues, within the second model the proportion of such signals can be evenly added to the existing directional choices. Probability of making a correct decision in a four-way choice situation such as encountered at the stem-stalk node by chance is 0.25 (Fig. 1a). In such situation, when males are making random directional choices, the behavioural threshold increased to 0.31 ms for FCS_1_ and 0.46 ms for FCS_2_ (Fig. 6a, green dashed line), despite the fact that 75% of such random directional choices was added to the wrong directions (Fig. 6a, orange dashed line).

Measurements revealed that *ΔA* was a correct directional cue only in 62% of analysed signals in the FCS bout. In the rest, the RMS amplitude was higher on the contralateral leaf stalk and according to our definition, these signals contained misleading cues. To adjust for the four-way choice at the branching, the first model tripled the contribution of misleading cues. As a result, the total proportion of signals containing correct cues was 35% and the probability of making a correct directional decision was no better than random at the theoretical threshold higher than 0.45 dB difference (Fig. 6c). Conversely, when projected to a four-way situation, the proportion of misleading cues was 65% but rapidly dropped below chance at −0.95 dB difference (Fig. 6c, solid orange line). In the second model, when ¼ of the sub-threshold information was added to the correct choice, the proportion of correct cues started at 31% of all signals and dropped to a chance level later, at 2.5 dB amplitude difference (Fig. 6d, dashed green line). However, this also resulted in adding 75% of sub-threshold cues to the wrong directional choices (Fig. 6c, solid blue line), making the total proportion of misleading cues rise up to 75% (Fig. 6d, dashed orange line), therefore making a wrong directional decision highly probable. Distribution of correct amplitude differences as a function of theoretical threshold did not intersect either of the lines that represent observed directional choices from behavioural trials in any implementation of the model.

## Discussion

Although it is now accepted that vibrational communication is not only the most widespread form of acoustic communication, but also that in evolutionary terms it is older than communication via air-borne sound^3,4,27^, many basic aspects of this communication system remain poorly understood. This is the first comprehensive study combining biophysical aspects of availability and reliability of directional cues, behavioural responses and neurophysiology in insects communicating with substrate vibrations.

The vibrational landscape of the signal active space on plants is complex, dependent on geometry and material properties of this heterogeneous substrate^14,18,28^; however, to gain a mating opportunity, males of *N*. *viridula* have to make fast and reliable directional choices during their search for the female. Our results show that the time difference of the signal’s arrival to spatially separated sensory inputs (i.e. legs) is a reliable directional cue. *Δt* depends on the distance between legs and signal propagation velocity, the latter depending on the type and physiological state of the plant (i.e. material properties of the transmission substrate) and, importantly, also on the signal’s frequency. Bending waves are the most important type of substrate vibrations used for communication on plants^18,29,30^. They have low propagation velocity, which also decreases with decreasing frequency. In this respect, vibrational signals of *N*. *viridula* with dominant frequencies between 80–120 Hz are well suited to create time differences large enough for the central nervous system to process. The calculated *Δt* for 200 Hz vibrational wave on *Vicia faba* plant across 1 cm distance is 0.26 ms^8^, which is within the range of measured time differences on *P*. *vulgaris* plant in the present study. Behavioural tests using two movable platforms that vibrate legs differentially revealed that 0.2 ms time difference was sufficient to enable directional response in a desert scorpion^23^, a termite^25^ and a leafcutter ant^31^, while in wandering spiders living on plants, the shortest time delay resulting in directional response was 2–4 ms^24,32^. In our behavioural experiments, males made fewer orientation errors in the presence of FCS_1_ than FCS_2._ It is possible that pulse trains with lower dominant frequency, and consequently lower propagation velocity and resulting larger *Δt*, provided a better cue for orientation. However, additional studies are needed to confirm this.

The *Δt* determined in the present study under natural conditions also had marked effect on the neuronal level. So far, all neurophysiological studies carried out on different insects revealed in the ventral cord a high number of interneurons responding to substrate vibrations^22,33–36^. In insects, vibration receptors located in the femoral chordotonal organs and subgenual organs project only to the ipsilateral part of their segmental ganglion^37,38^ and integration of the inputs from all six legs is performed by intra- and intersegmental interneurons. Such vibratory interneurons have been physiologically and morphologically described in the field cricket (*Gryllus campestris*) relying primarily on air-borne sound^8^, non-hearing cave-crickets^36,39^, as well as in *N*. *viridula*^22^. However, the integration of inputs from six legs at the level of the central nervous system has been poorly studied^8^. In the migratory locust (*Locusta migratoria*), some ascending interneurons showed direction-dependent response patterns when different leg pairs were stimulated with a time delay at threshold values between 1–3 ms^40^, while in *G*. *campestris* some interneurons showed markedly different response pattern when the temporal pattern of leg stimulation was changed and time difference of 0.75 ms was used^8^. The interneurons stained in the present work are mostly similar to those described previously, effectively connecting the input region of a single leg within the ganglion with three contralateral legs^22^. Described interneurons featured smooth-like dendrites associated with the input area in one region of the ganglion and beaded dendrites indicating output areas in other regions in the central nervous system^41^. Conveying the information of leg stimulation to the contralateral side is mandatory to process directionality. A computational model of orientation in desert scorpion, based on anatomical work in the wandering spider *Cupiennius salei*^42,43^, proposed a network in which each leg recieves a summed inhibition from three other legs^44^. Ring-like neurons similar to plurisegmental bilateral type-III neuron described in wandering spider^43^ were not found in stink bugs, yet it is clear that interneurons receiving input from a single leg convey the information to regions related to other legs as well^22^.

Behavioural studies with movable platforms also showed that desert scorpions^23^ and wandering spiders^24^ can use amplitude difference for orientation and in the latter, the lowest amplitude difference resulting in directional response was 10 dB. The leg span of adult wandering spiders is around 10 cm and such amplitude difference corresponds to the amplitude difference created across such distance under natural conditions on the *Agave americana* plant^32^. However, the present study showed that, in contrast to *Δt*, the amplitude difference (measured as differences of RMS amplitude) between the ipsilateral and contralateral leaf stalk is an unreliable directional cue for the much smaller *N*. *viridula* standing on *P*. *vulgaris*. Although the true amplitude difference for FCS could be as high as 3.5 dB, the amplitude was often higher on the contralateral stalk. The plant was revealed as the most important factor influencing the reliability of *ΔA* as a directional cue. One possible explanation for such high unpredictability of this directional cue is heterogeneity of plant structure. Wave propagation differs between parts with different mechanical properties, like for example stiffness and thickness^11,18,29^. Moreover, due to the plant resonant properties, wave reflections and frequency-dependent standing-wave pattern of vibrations, the amplitude of vibrational signal on plants oscillates with distance from the source^18–20^. Changes in impedance at nodes or veins can act as a source of reflected waves, which are then superimposed on the original signal and change its properties such as envelope shape and duration^18,45^. The position of the source of vibration also had an important effect on reliability of *ΔA*_*true*_. It is conceivable that due to differences in transmission properties, the pyhsical characteristics of the plant at the position of the signaller have an additional effect on signal transmission.

Although in playback tests, vibrational signal itself was revealed as the least important factor influencing the reliability of *ΔA*_*true*_ as a directional cue, the structure of the signal is likely to be important when emitted by a live female. We observed that the side of higher amplitude was changing even within one calling bout of a stationary female; however, such changes were not observed when pure tone vibrational signals were applied to the leaf. A factor influencing the signal structure and, consequently, its propagation, is the signal production mechanism. In *N*. *viridula*, vibrations of the abdomen produce vibrational pulse trains. These vibrations are transmitted to the plant via the legs^16^. Muscles associated with this signal production mechanism contract synchronously and each contraction cycle results in one oscillatory wave of the abdomen^16,17^. Since muscle action are directly related to the frequency of the emitted vibrational pulse train, any change in muscle activity during the production of each pulse train within the calling bout results in changed frequency structure of the signal, which in turn, results in different propagation properties. Different pulse train durations may also change the superposition of the signal with its reflections and thus change amplification into attenuation or vice-versa. Moreover, the frequency structure of the pulse train may also depend on the coupling between the signalling female and the plant, in particular the leg stiffness^18,46^, which can change even if the calling female does not move.

While the present study shows that under natural conditions encountered on plants by *N*. *viridula*, amplitude difference is not a directional cue that enables accurate and fast location of the female, this cue may provide more reliable directional information for other, especially larger arthropods living on other substrates. However, it should be mentioned that when sand scorpions were presented with conflicting cues (earlier, weaker vs. later, stronger), they always turned towards the side that moved first^23^. It should also be emphasized that on plants, insect’s legs are often positioned in a three-dimensional array around the stem and that under natural conditions, vibrational signals arrive at different legs at different times and with different amplitudes. In *L*. *migratoria*, some of the recorded interneurons showed improved directional response when amplitude difference simulating the position of vibratory source in the front or behind the locust was applied in addition to time difference^40^.

The decision model used in the present study integrated the data from signal transmission of a naturally emitted FCS and provided a useful tool for testing various hypotheses against the data from behavioural and neurophysiological experiments, which could not be obtained simultaneously. With it, we confirmed that directional choices made by males are in agreement with availability of directional cues represented by *Δt*, coupled with the capacity of neuronal system to resolve such small delays. Based on our data, the model also suggests that the range of the “true” neuronal threshold should be between 0.31 ms and 0.46 ms, both lower than our tested value of 0.5 ms. The two tested paradigms – male waiting for an above-threshold cue or making a random directional decision in case of a sub-threshold cue – provided similar results. On the other hand, *ΔA* proved unreliable, regardless of the neuronal system’s capacity. Under no paradigm and no hypothetical threshold could the male make a directional decision with more than 35% probability of choosing the branch leading to the signal source, which is in disagreement with the observed orientation behaviour.

In summary, our study shows that the difference in arrival times of vibrational wave reaching vibration receptors located in the legs is the most likely cue underlying directional behaviour in *N*. *viridula*. Precise determination of behavioural and neurophysiological thresholds remains open, although current data suggests that 0.5 ms time delay between inputs is both available and sufficient directional cue. These findings contribute to general understanding of orientation mechanisms in small animals; however, they are also important in the light of recent developments in behavioural manipulation in pest control^47^, where information about how insects utilize available cues is crucial in development of vibration-based solutions. One example is the on-going development of vibration-mediated mass trapping of the invasive brown marmorated stink bug (*Halyomorpha halys*)^48^, where care should be taken in trap construction to provide correct cues. Furthermore, the findings can be used to develop biomimetic solutions in the field of miniature robotics.

## Materials and Methods

### Experimental animals

All experiments were done on sexually mature *Nezara viridula* (L.) (Hemiptera: Pentatomidae) from a single Slovenian population at the North Adriatic coastal region. Experimental animals, aged 10–20 days after the final moult, were obtained from the laboratory colony kept at the National Institute of Biology, Ljubljana, Slovenia.

### Male searching behaviour

To obtain the information on the first male directional choice at the junction between the main stem and two leaf stalks we analysed the video recordings of experiments described previously^13,49^. In brief, a single male was placed on the leaves at the apex of the potted bean (*Phaseolus vulgaris*) plant in order to exclude negative geotaxis influencing his choice. After 2 minutes of acclimation period, the male was presented with 13 minutes of playback stimulation with female calling song (FCS). We applied vibratory stimulus to the right or left leaf and the side from which the stimulation was applied was randomly changed. Stimulatory FCS and male vibrational responses were recorded with laser vibrometer (Polytec OFV 2200 with 353 controller-head) and stored onto a hard drive using Cool Edit Pro 2 (Syntrillium Software, USA). Male behaviour together with vibrational signals was filmed with the video camera.

Female calling songs used for stimulation consisted of unmodified natural sequences of pulse trains within the calling bout recorded on the middle-tone loudspeaker membrane in order to eliminate the filtering effect of the substrate. Since the FCS parameters of Slovenian population can differ between years^15^, FCSs used for stimulation were emitted by females collected in the same year as males used in orientation experiments and the spectral characteristics and temporal parameters had mean values that corresponded to the preferred values of males collected that year (FCS_1_: pulse train duration = 1621 ± 187 ms; pulse train repetition time 5155 ± 701 ms; dominant frequency = 83 Hz; FCS_2_^13^: pulse train duration = 700 ± 10 ms; pulse train repetition time 2610 ± 20 ms; dominant frequency = 116 Hz)^15,49^. Eleven males were tested with FCS_1_^13^ and 19 with FCS_2_^49^.

The amplitude of stimulation was adjusted to the level of natural signals. We monitored the first directional choice the male made at the branching point. A Chi-square test for contingency tables was used to compare the numbers of males choosing first the vibrated branch with the theoretical choice under random situation (i.e. in the absence of FCS). In such situation with a four-way crossing at the branching point the probability that a male would randomly choose the correct branch is 0.25.

### Transmission of vibration signals over the stem-stalk node

#### Propagation of naturally emited FCS

Propagation of vibrational signals across the stem-stalk branching was tested using sexually mature *N*. *viridula* females placed on a bean leaf and the emission of FCS was induced by the proximity of a sexually mature male not in contact with the plant. The *P*. *vulgaris* plants were grown from the seeds in the laboratory and used after two weeks, when the first branching node (two opposite leaves) was fully developed and the plants were approximately 15 cm tall.

Five different plants were used. FCS pulse trains were recorded simultaneously with two PDV 100 laser vibrometers (Polytec, GmbH, Waldbronn, Germany) directed perpendicularly at a point on each leaf stalk 5 mm away from the branching (Fig. 2a), which matches the maximum leg span in *N*. *viridula*^8^. A piece of reflective tape was attached to each recording point to improve reflection. The vibrometers registered vibrations in the laser beam plane and were connected to a computer sound card (Sound Blaster Audigy 4, Creative Labs Inc). Each vibrometer’s output was recorded in its own channel at 48 kHz sample rate. Thus, a single channel contained vibrations registered from either ipsilateral or contralateral side of the branching relative to the position of the calling female. The two channels were synchronized and calibrated against each other for the amplitude. Amplitude was recorded in sample values, relative to the maximum sampling capability of the recording equipment. Recordings were stored in an uncompressed wave format on the computer hard drive for subsequent analysis.

The recordings were filtered using the CoolEdit Pro’s Noise reduction function. We analysed five randomly chosen consecutive pulse trains in a calling bout of each of 11 females. The phase velocity delay of the bending waves was measured with a custom function, written in Python 3 (Python Software Foundation, USA). The function computed delays using cross-correlation on the rectified signal: delay value was determined at maximum cross-correlation value. Amplitude comparison of the pulse train registered on the ipsilateral and contralateral stalk was made with another custom written function, which computed the differences between RMS amplitudes and expressed it in dB.

#### Two−dimensional characterization of FCS propagation

Since registration of stalk vibration with one laser vibrometer may underestimate the true amplitude of vibration^11,26^, two PDV-100 laser vibrometers were added to the setup described above. Two laser vibrometers were used per stalk, positioned perpendicularly to the stem and to each other (Fig. 3a). Outputs from the four laser vibrometers were recorded on four channels of the calibrated data acquisition device Sinus Soundbook Quadro running Sinus Samurai 2.6 software (Sinus Messtechnik GmbH, Leipzig, Germany) which were set to 52 kHz sampling rate. The laser vibrometers were synchronized and calibrated against each other. Such setup allowed us to compute the true amplitude (*A*_*true*_)^11,26^. Briefly, the amplitudes acquired at the same position at same time (*t*) were used as *X*, *Y* coordinates in the Cartesian coordinate system. For each stalk, true amplitude was obtained via conversion to polar coordinates, where the true amplitude value is represented as a distance from the origin to the point determined by X, Y pair (Fig. 3a, inset). Difference in RMS of *A*_*true*_ and time delay were calculated in the same way as described above. Recordings that contained background noise in any channel were excluded from further analyses due to the potential impact on cross-correlation.

In these tests, five different bean plants were used. In each of them, stimuli were delivered at four different locations on the leaf (base, midpoint, apex and side of the lamina) (Fig. 3a) by a minishaker (Type 4810, Brüel & Kjær, Nærum, Denmark). We used three different stimuli. 100 Hz and 150 Hz sine wave signals were synthesized in Audacity 2.1.3 (Audacity Team) and individual signals (duration 1 s, 0.1 ms rise and fall time) were composed into a sequence including five signals of each frequency (signal repetition time = 2 s). FCS applied to the leaf consisted of 10 consecutive pulse trains from a calling bout of a female recorded from the centre of the membrane of the middle-tone loudspeaker (pulse train duration = 958 ± 65 ms; pulse train repetition time = 3743 ± 458 ms; dominant frequency = 111 ± 3 Hz). We adjusted the source amplitude of all stimuli to match the natural range of female calls.

A classifier named “RandomForestClassifier” from Python’s Scikit-Learn machine-learning package^50^ was used to determine the importance of different parameters (bean plant, stimulus delivery location and stimulus type) on reliability of *ΔA*_*true*_ as a directional cue. We prepared the data by converting *ΔA*_*true*_ values into categorical “correct” and “wrong”. The classifier was trained on the three parameters with 50 iterations with bootstrapping. The averaged parameter importance (as a relative score) was extracted from classifier together with the standard deviation of the mean. Briefly, the algorithm draws dichotomous decision trees that predict the value of a target variable by finding the categorical feature that will yield the largest information gain for categorical targets for each node, yielding a set of simple decision rules. Subsamples are obtained by bootstrapping and decision trees obtained are averaged. Parameter importance score is considered as a weight of the parameter, contributing to the best tree^50^.

### Neurophysiology

In total, 42 males were used in neurophysiological experiments. Animals were waxed dorsal side up on a U-shaped holder with legs freely dangling down. Thoracic part of central nervous system was exposed from dorsal side by removing the scutellum, pronotum and gut. The holder was then placed above two minishakers (Type 4810; Brüel & Kjær, Denmark), which delivered the stimuli. Legs were attached to custom-made platforms fastened to minishakers’ heads in natural-like position. Two situations with three different combinations of legs were tested (Fig. 4a): left – right (stimulating prothoracic left/prothoracic right or metathoracic left/metathoracic right legs) and diagonal (stimulating prothoracic right/metathoracic left legs). Other legs were removed. Intracellular recordings were made from either prothoracic (PTG) or central ganglion (CG). Data acquisition was performed with CED 1401*plus* and Spike2 v5 (CED, Cambridge, UK). Microelectrodes had resistance between 80–100 MΩ. We used a laser vibrometer to ensure there was no vibratory coupling between the two minishakers. Either Lucifer Yellow dye (5% in 0.5 M LiCl) or Alexa Fluor 568 (10 mM in 200 mM KCl) was injected in attempt to obtain staining.

Playback stimuli were generated with CoolEdit Pro and were fed to the minishakers from a computer sound card via custom-made power amplifiers. Stimuli were 100 ms long sine waves with 10 ms rise and fall phases. 150 Hz sine wave was taken as a reasonable approximation of *N*. *viridula*’s natural signal^15^. The stimuli were initially presented with signal velocity of 5 × 10^−4^ m/s. The legs were repeatedly shaken during the electrode penetration. After a neuron that showed response to the vibratory stimulation at 150 Hz was encountered, we tested its sensitivity to time delays in the onset of stimulation of the two selected legs. The time delays used were 0.5 and 1.0 ms with either one or the other leg being delayed; together with a control (0.0 ms) where there was no delay, each interneuron was tested with five different stimuli.

Neuronal recordings were exported from Spike2 for quantization. Response latency and spike count were evaluated using custom written code in Python 3. Spikes were counted and latency measured in the time window from 0.00–0.12 s after the beginning of the stimuli. In all interneurons in which time delay had an impact on neuronal activity, the effect of a delay to one of the legs was more pronounced and we labelled this leg as dominant, while the other was named subdominant.

The central nervous system was processed using standardized procedure, described before^22^. Whole-mount preparations were photographed and stitched together to assist tracing. Tracing was done using Adobe Illustrator CS.

For clarity and a display purpose only, in Figs 4 and 5, physiological responses and morphology of some interneurons are shown as left-right mirror images in order to correspond to the dominant-subdominant legs (sides) of the other interneurons.

### Model predictions

Distribution of measured *Δt* and *ΔA* shown in Fig. 2b was fitted with Gaussian function (Eq. 1); *A* is the scaling factor, *x* the measured value, *μ* the mean value and *σ*^2^ the variance.

1 $$P=A{e}^{-\frac{{(x-\mu )}^{2}}{2{\sigma }^{2}}}$$

The first model assumed that a male takes no action when receiving subthreshold cues. Proportion of *Δt* cues indicating the correct direction was then computed as complementary cumulative distribution function (CDF) to the Gaussian fit of the distribution (Eq. 1) from hypothetical *t*_*threshold*_ onwards (Eq. 2):

2 $${P}_{correct\_dt}=1-\frac{1}{\sigma \sqrt{2\pi }}{\int }_{{t}_{threshold}}^{t}{e}^{\frac{-{(t-\mu )}^{2}}{2{\sigma }^{2}}}dt$$

Proportion of subthreshold time delays was computed as CDF to the Gaussian fit of the distribution (Eq. 1) up to the hypothetical *t*_*threshold*_ (Eq. 3):

3 $${P}_{subthreshold\_dt}=1-\frac{1}{\sigma \sqrt{2\pi }}{\int }_{t}^{{t}_{threshold}}{e}^{\frac{-{(t-\mu )}^{2}}{2{\sigma }^{2}}}dt$$

The second model assumed that a male makes a random choice when exposed to a subthreshold time delay cue. There are four possibilities on the node and we assumed that each is equally likely to be chosen by the searching male in absence of above-threshold cues. Because the experimental data was limited to two nodes of the crossing out of four, we added one quarter of subthreshold *dt* (*P*_*subthreshold_dt*_) to the proportion of correct *Δt* (*P*_*correct_dt_with_random*_), which was computed point-wise at the value of the theoretical threshold (Eq. 4). Conversely, we added three quarters of *P*_*subthreshold_dt*_ to the proportion of misleading *Δt* (*P*_*wrong_dt_with_random*_) (Eq. 5).

4 $${P}_{correct\_dt\_with\_random}=\,{P}_{correct\_dt}+\,0.25\,{P}_{subthreshold\_dt}$$

5 $${P}_{wrong\_dt\_with\_random}=\,0.75\,{P}_{subthreshold\_dt}$$

As for *Δt*, the proportion of correct amplitude differences (*ΔA*) was computed as CDF, using the parameters obtained in the fit of distribution with the Gaussian from Eq. 1. Measuring signal propagation showed that amplitude difference provided correct directionality information in 62% of signals and incorrect in 38%. After adjusting to a four-way situation as above, the proportion of correct *ΔA* against misleading *ΔA* stood at 35% against 65%. These weights were then used in computing the proportion of correct *ΔA* (*P*_*correct_dA*_) and proportion of misleading *ΔA* (*P*_*wrong_dA*_). *P*_*correct_dA*_ was computed as CDF, using parameters obtained in the fit of distribution with the Gaussian from Eq. 1 (Eq. 6) and *P*_*wrong_dA*_ was computed as complementary CDF (Eq. 7).

6 $$({P}_{correct\_dA}|\,{A}_{threshold}\ge 0\,)=0.35(1-\frac{1}{\sigma \sqrt{2\pi }}{\int }_{{A}_{threshold}}^{A}{e}^{\frac{-{(A-\mu )}^{2}}{2{\sigma }^{2}}}dA)$$

7 $$({P}_{wrong\_dA}|\,{A}_{threshold}\le 0\,)=0.65(\frac{1}{\sigma \sqrt{2\pi }}{\int }_{A}^{{A}_{threshold}}{e}^{\frac{-{(A-\mu )}^{2}}{2{\sigma }^{2}}}dA)$$

Similarly, the subthreshold *ΔA* (*P*_*subthreshold_dA*_) was computed as CDF in case of correct *ΔA* and as complementary CDF in case of misleading *ΔA*.

8 $$({P}_{subthreshold\_dA}|\,{A}_{threshold}\ge 0\,)=0.35(\frac{1}{\sigma \sqrt{2\pi }}{\int }_{{A}_{threshold}}^{A}{e}^{\frac{-{(A-\mu )}^{2}}{2{\sigma }^{2}}}dA)$$

9 $$({P}_{subthreshold\_dA}|\,{A}_{threshold}\le 0\,)=\,0.65(1-\frac{1}{\sigma \sqrt{2\pi }}{\int }_{A}^{{A}_{threshold}}{e}^{\frac{-{(A-\mu )}^{2}}{2{\sigma }^{2}}}dA)$$

Again, the second model split the sub-threshold signals assuming random choice (0.25/0.75) as shown in Eqs 10 and 11.

10 $${P}_{correct\_dA\_with\_random}=\,{P}_{correct\_dA}+\,0.25\,{P}_{subthreshold\_dA}$$

11 $${P}_{wrong\_dA\_with\_random}={P}_{wrong\_dA}+0.75\,{P}_{subthreshold\_dA}$$

### Data availability

The datasets generated and/or analysed during the current study are available from the corresponding author on reasonable request.

## Electronic supplementary material

Supplementary Video S1
